# Supplementary material for: Comparative anatomical and transcriptomic analyses of the color variation of leaves in Aquilaria sinensis
Source: PeerJ. 2021 Jun 22;9:e11586. doi: 10.7717/peerj.11586 (PMC8231315; doi:10.7717/peerj.11586)
Supplement: Supplemental Information 1 [file peerj-09-11586-s001.docx]

**Table S1. DNA barcodes used in phylogenetic tree construction**

| DNA barcode locus | Name | Gene accession |
| --- | --- | --- |
| ITS2 | GS | MT548012, MT548013, MT548014 |
|  | NS | MT548015, MT548016, MT548017 |
|  | *A. crassna* | KU244108, KU244109, KU244110, KU244111 |
|  | *A. hirta* | KU244112, KU244113, KU244114 |
|  | *A. malaccensis* | KU244115, KU244116, KU244117 |
|  | *A. microcarpa* | KU244118, KU244119, KU244120 |
|  | *A. sinensis* | KU244121, KU244122, KU244123, KU244124, KU244125, KU244126 |
|  | *A. subintegra* | KU244127, KU244128 |
|  | *A. yunnanensis* | KU244129, KU244130, KU244131 |
|  | *Gyrinops versteegii* | KU244132 |
|  | *Gonystylus bancanus* | KU244133 |
| *trn*L-*trn*L | GS | MT548018, MT548019, MT548020 |
|  | NS | MT548021, MT548022, MT548023 |
|  | *A. crassna* | KU244030, KU244031, KU244032, KU244033 |
|  | *A. hirta* | KU244034, KU244035, KU244036 |
|  | *A. malaccensis* | KU244037, KU244038, KU244039 |
|  | *A. microcarpa* | KU244040, KU244041, KU244042 |
|  | *A. sinensis* | KU244043, KU244044, KU244045, KU244046, KU244047, KU244048 |
|  | *A. subintegra* | KU244049, KU244050 |
|  | *A. yunnanensis* | KU244051, KU244052, KU244053 |
|  | *Gyrinops versteegii* | KU244054 |
|  | *Gonystylus bancanus* | KU244055 |
