# Supplementary material for: Comparative anatomical and transcriptomic analyses of the color variation of leaves in Aquilaria sinensis
Source: PeerJ. 2021 Jun 22;9:e11586. doi: 10.7717/peerj.11586 (PMC8231315; doi:10.7717/peerj.11586)
Supplement: Supplemental Information 2 [file peerj-09-11586-s002.docx]

**Table S2. Anatomical parameters of the transverse section of leaves**

| Item | LGS | LNS | P value |
| --- | --- | --- | --- |
| Total area of IP in midrib (μm^2^) | 8074.67 ± 968.79 | 1503.45 ± 169.18 | <0.0001 |
| PT thickness (μm) | 26.18 ± 2.87 | 30.12 ± 3.24 | <0.0001 |
| ST thickness (μm) | 104.81 ± 10.52 | 91.50 ± 6.55 | <0.0001 |
| The ratio of PT to ST | 0.25 | 0.33 | Null |
| UE cell thickness (μm) | 13.24 ± 1.76 | 13.92 ± 1.78 | 0.0475 |
| LE cell thickness (μm) | 8.90 ± 1.67 | 9.11 ± 1.43 | 0.5712 |

Note: P value indicates the significance of the statistical difference between the measured items of LGS and LNS. P value < 0.05 suggests the significant statistical difference. (IP: included phloem, PT: palisade tissue, ST: spongy tissue, UE: upper epidermis cell, LE: lower epidermis cell)
