# Supplementary material for: Comparative anatomical and transcriptomic analyses of the color variation of leaves in Aquilaria sinensis
Source: PeerJ. 2021 Jun 22;9:e11586. doi: 10.7717/peerj.11586 (PMC8231315; doi:10.7717/peerj.11586)
Supplement: Supplemental Information 3 [file peerj-09-11586-s003.docx]

**Table S3. Anatomical parameters of the transverse section of stems**

| Item | SGS | SNS | P value |
| --- | --- | --- | --- |
| Single IP area (μm^2^) | 1750.41 ± 992.35 | 4794.15 ± 1547.28 | <0.0001 |
| Single IP length (μm) | 82.65 ± 29.21 | 127.27 ± 37.52 | <0.0001 |
| Single IP width (μm) | 31.16 ± 10.41 | 56.02 ± 12.33 | <0.0001 |
| Percentage of IP in xylem (%) | 19.72 ± 2.77 | 21.36 ± 2.23 | 0.0075 |
| Single Ve area (μm^2^) | 173.55 ± 105.67 | 286.45 ± 132.61 | <0.0001 |
| Feret diameter along major axis of single Ve (μm) | 14.16 ± 4.15 | 22.08 ± 4.56 | <0.0001 |
| Feret diameter along minor axis of single Ve (μm) | 10.51 ± 3.53 | 15.87 ± 4.16 | <0.0001 |
| Percentage of Ve in xylem (%) | 6.35 ± 1.15 | 7.74 ± 1.26 | <0.0001 |
| Single WF area (μm^2^) | 19.57 ± 9.32 | 72.67 ± 37.39 | <0.0001 |
| Feret diameter along major axis of single WF (μm) | 4.39 ± 1.07 | 9.39 ± 2.67 | <0.0001 |
| Feret diameter along minor axis of single WF (μm) | 3.07 ± 0.80 | 6.28 ± 1.63 | <0.0001 |

Note: P value indicates the significance of the statistical difference between the measured items of LGS and LNS. P value < 0.05 suggests the significant statistical difference. (IP: included phloem, Ve: vessel, WF: wood fiber cell)
