# Supplementary material for: Comparative anatomical and transcriptomic analyses of the color variation of leaves in Aquilaria sinensis
Source: PeerJ. 2021 Jun 22;9:e11586. doi: 10.7717/peerj.11586 (PMC8231315; doi:10.7717/peerj.11586)
Supplement: Supplemental Information 4 [file peerj-09-11586-s004.docx]

**Table S4. Summary of sequencing reads**

| Sample | Paired-end | Total reads | Total bases | Sequence length (nt) | GC content (%) | Encoding |
| --- | --- | --- | --- | --- | --- | --- |
| LNS_1 | Read1 | 23,112,809 | 3,411,734,114 | 150 | 47 | Sanger / Illumina 1.9 |
|  | Read 2 | 23,112,809 | 3,411,757,718 | 150 | 47 |  |
| LNS_2 | Read 1 | 19,585,485 | 2,937,822,750 | 150 | 47 |  |
|  | Read 2 | 19,585,485 | 2,937,822,750 | 150 | 47 |  |
| LNS_3 | Read 1 | 17,753,982 | 2,663,097,300 | 150 | 47 |  |
|  | Read 2 | 17,753,982 | 2,663,097,300 | 150 | 46 |  |
| LGS_1 | Read 1 | 15,047,933 | 2,257,189,950 | 150 | 49 |  |
|  | Read 2 | 15,047,933 | 2,257,189,950 | 150 | 49 |  |
| LGS_2 | Read 1 | 14,847,670 | 2,227,150,500 | 150 | 46 |  |
|  | Read 2 | 14,847,670 | 2,227,150,500 | 150 | 47 |  |
| LGS_3 | Read 1 | 18,536,509 | 2,780,476,350 | 150 | 47 |  |
|  | Read 2 | 18,536,509 | 2,780,476,350 | 150 | 47 |  |
| SNS_1 | Read 1 | 18,876,082 | 2,782,214,684 | 150 | 47 |  |
|  | Read 2 | 18,876,082 | 2,782,243,263 | 150 | 47 |  |
| SNS_2 | Read 1 | 16,805,969 | 2,489,788,123 | 150 | 47 |  |
|  | Read 2 | 16,805,969 | 2,489,807,816 | 150 | 47 |  |
| SNS_3 | Read 1 | 19,547,359 | 2,932,103,850 | 150 | 47 |  |
|  | Read 2 | 19,547,359 | 2,932,103,850 | 150 | 47 |  |
| SGS_1 | Read 1 | 18,787,725 | 2,773,275,294 | 150 | 48 |  |
|  | Read 2 | 18,787,725 | 2,773,270,431 | 150 | 48 |  |
| SGS_2 | Read 1 | 17,131,356 | 2,569,703,400 | 150 | 49 |  |
|  | Read 2 | 17,131,356 | 2,569,703,400 | 150 | 49 |  |
| SGS_3 | Read 1 | 18,655,143 | 2,798,271,450 | 150 | 50 |  |
|  | Read 2 | 18,655,143 | 2,798,271,450 | 150 | 50 |  |
| Total | | 437,376,044 | 65,245,722,543 |  |  |  |
