# Supplementary material for: Comparative anatomical and transcriptomic analyses of the color variation of leaves in Aquilaria sinensis
Source: PeerJ. 2021 Jun 22;9:e11586. doi: 10.7717/peerj.11586 (PMC8231315; doi:10.7717/peerj.11586)
Supplement: Supplemental Information 5 [file peerj-09-11586-s005.docx]

**Table S5. Stats for all *de novo* assembled transcript contigs**

| Item | Value |
| --- | --- |
| Total trinity genes | 26,381 |
| Total trinity transcripts | 62,907 |
| GC content (%) | 42.8 |
| Contig N10 (nt) | 4,833 |
| Contig N20 (nt) | 3,768 |
| Contig N30 (nt) | 3,112 |
| Contig N40 (nt) | 2,588 |
| Contig N50 (nt) | 2,168 |
| Median contig length (nt) | 1,035 |
| Average contig length (nt) | 1,417.90 |
| Total assembled bases | 89,198,112 |
