# Supplementary material for: Comparative anatomical and transcriptomic analyses of the color variation of leaves in Aquilaria sinensis
Source: PeerJ. 2021 Jun 22;9:e11586. doi: 10.7717/peerj.11586 (PMC8231315; doi:10.7717/peerj.11586)
Supplement: Supplemental Information 6 [file peerj-09-11586-s006.docx]

**Table S6. Gene Ontology enrichment analysis for differentially expressed genes downregulated in LGS compared to LNS**

| Ontology | Term | Gene number | FDR |
| --- | --- | --- | --- |
| Biological process | response to abscisic acid | 16 | 0.0341 |
|  | fruit ripening | 4 | 0.0341 |
|  | ethylene biosynthetic process | 4 | 0.0341 |
|  | recognition of pollen | 8 | 0.0341 |
|  | response to aluminum ion | 3 | 0.0341 |
|  | defense response | 23 | 0.0341 |
|  | oxidation-reduction process | 28 | 0.0430 |
| Cellular component | plasmodesma | 25 | 0.0139 |
|  | plant-type vacuole | 6 | 0.0178 |
| Molecular function | xenobiotic transmembrane transporting ATPase activity | 6 | 0.0005 |
|  | ADP binding | 15 | 0.0005 |
|  | ATPase activity, coupled to transmembrane movement of substances | 9 | 0.0072 |
