# Supplementary material for: Comparative anatomical and transcriptomic analyses of the color variation of leaves in Aquilaria sinensis
Source: PeerJ. 2021 Jun 22;9:e11586. doi: 10.7717/peerj.11586 (PMC8231315; doi:10.7717/peerj.11586)
Supplement: Supplemental Information 7 [file peerj-09-11586-s007.docx]

**Table S7. Gene Ontology enrichment analysis for differentially expressed genes downregulated in SGS compared to SNS.**

| Ontology | Term | Gene number | FDR |
| --- | --- | --- | --- |
| Biological process | nitrate transport | 3 | 0.0187 |
| Molecular function | ADP binding | 8 | 0.0042 |
|  | sucrose alpha-glucosidase activity | 3 | 0.0200 |
|  | double-stranded RNA binding | 3 | 0.0239 |
|  | protein dimerization activity | 6 | 0.0239 |
|  | oxidoreductase activity | 9 | 0.0239 |
|  | RNA helicase activity | 2 | 0.0239 |
|  | glutamate-ammonia ligase activity | 2 | 0.0239 |
|  | RNA-directed 5'-3' RNA polymerase activity | 2 | 0.0304 |
