# Supplementary material for: Comparative anatomical and transcriptomic analyses of the color variation of leaves in Aquilaria sinensis
Source: PeerJ. 2021 Jun 22;9:e11586. doi: 10.7717/peerj.11586 (PMC8231315; doi:10.7717/peerj.11586)
Supplement: Supplemental Information 8 [file peerj-09-11586-s008.docx]

**Table S8. Screened PR genes in differentially expressed genes.**

| Gene ID | Gene symbol | Family | Log2FC_a_ | FDR_a_ | Log2FC_b_ | FDR_b_ |
| --- | --- | --- | --- | --- | --- | --- |
| E_H239278_c0_g1 | PRB1 | PR1 | -5.2072 | 0.003 |  |  |
| E_H268278_c2_g4 | PRR1 | PR1 | -4.4122 | 0.0286 |  |  |
| E_H229178_c0_g1 | PR-1 | PR1 | -4.0426 | 0.0091 |  |  |
| E_H252747_c0_g1 | GNS1 | PR2 | -9.8857 | <0.0001 |  |  |
| E_H252747_c0_g2 | E13B | PR2 | -6.9149 | <0.0001 |  |  |
| E_H249895_c0_g2 | E133 | PR2 | -6.1953 | <0.0001 |  |  |
| E_H252145_c0_g4 | E136 | PR2 | -3.5602 | 0.0079 |  |  |
| E_H248541_c0_g1 | E137 | PR2 | -3.332 | 0.027 |  |  |
| E_H261617_c0_g2 | E1313 | PR2 | -3.2493 | 0.0233 |  |  |
| E_H261526_c2_g1 | E1311 | PR2 | -2.3579 | 0.0038 |  |  |
| E_H265619_c1_g1 | E1314 | PR2 | 3.174 | 0.0014 |  |  |
| E_H264156_c1_g3 | TBR | PR4 | -3.0307 | 0.0125 |  |  |
| E_H268278_c1_g1 | TLP | PR5 | -4.9315 | 0.0022 |  |  |
| E_H250019_c0_g1 | TLP1 | PR5 |  |  | -2.8041 | 0.05 |
| E_H256801_c4_g1 | PER3 | PR9 | -5.0499 | 0.0017 |  |  |
| E_H262250_c2_g1 | PNC2 | PR9 | -3.9717 | 0.0343 |  |  |
| E_H258413_c4_g1 | PER47 | PR9 | -3.7575 | 0.0047 |  |  |
| E_H261201_c0_g1 | PER11 | PR9 |  |  | -5.4678 | 0.0001 |
| E_H225675_c0_g1 | PER21 | PR9 |  |  | 7.9589 | 0.0032 |
| E_H236209_c0_g1 | STH-2 | PR10 | -4.4692 | 0.0027 |  |  |
| E_H267359_c0_g3 | STH-21 | PR10 |  |  | 3.2225 | 0.0332 |
| E_H262110_c1_g1 | NLTP | PR14 | -7.187 | 0.0001 |  |  |
| E_H265005_c0_g2 | LTP4 | PR14 | -2.7736 | 0.02 |  |  |
| E_H260494_c1_g4 | LTP8 | PR14 |  |  | -5.3878 | 0.0014 |
| E_H259179_c0_g1 | GL116 | PR15 | -5.8398 | 0.0372 | -3.9639 | 0.0039 |

Note: Negative Log2FC represents the upregulated genes in GS while the positive value represents the downregulated genes. High absolute value of Log2FC represents a high fold change value. FDR is false discovery rate suggesting the reliability. The letter “a” beside “Log2FC” or “FDR” represents the value derives from leaves while “b” represents stems. The blank lattices suggest no significant difference.
