# Supplementary material for: Comparative anatomical and transcriptomic analyses of the color variation of leaves in Aquilaria sinensis
Source: PeerJ. 2021 Jun 22;9:e11586. doi: 10.7717/peerj.11586 (PMC8231315; doi:10.7717/peerj.11586)
Supplement: Supplemental Information 9 [file peerj-09-11586-s009.docx]

**Table S9. Primers used in qRT-PCR**

| Gene ID | Gene symbol | Primer | |
| --- | --- | --- | --- |
| E_H258264_c2_g1 | LHCB1(1) | F: CCACCACCAAGCAATCAGG |  |
|  |  | R: GTGTCCCAACCATAGTCACCAG |  |
| E_H256684_c0_g2 | LHCB4 | F: GACGGGAGTTTAGTGGGTGATT |  |
|  |  | R: ACGTTCTTCGCCAGGTTCTG |  |
| E_H263330_c2_g2 | LHCB6 | F: CATTGGCAAAGTTCTCAGCG |  |
|  |  | R: ACTTCTGGGGACTCAGCTCCT |  |
| E_H267499_c3_g5 | LHCB2 | F: TAAGCCAACAAGTAGGCGTCG |  |
|  |  | R: TGGGGCGGTTTTGACAGT |  |
| E_H258264_c1_g1 | LHCB1(2) | F: GCCGCCTTCTGAGAAGATCT |  |
|  |  | R: CCAGGAACGGCGTCAAGTT |  |
| E_H263776_c0_g1 | PSBR | F: GAAGGAAGGGCAAGGGAAAG |  |
|  |  | R: GCCACCAACGTAAACATCACC |  |
| E_H249053_c0_g2 | PSAO | F: ATCCTCGGAGACCTGGAAGG |  |
|  |  | R: GTGATCGCTGGAGGAGTTGG |  |
| E_H263876_c1_g1 | PABO | F: TGCTCTGGATTTCGTCGTAGG |  |
|  |  | R: TTAAGGACTTGGCTCAAAGGTG |  |
| E_H252989_c0_g1 | CLH1 | F: CCGGAAATACTCACTCACAAAGAC |  |
|  |  | R: TTGTCACCCTTCCCACTCTTG |  |
| E_H265995_c2_g2 | CHLP | F: GATGCTCGGGGATTGGATG |  |
|  |  | R: CGGATTTCTACGGTTGGGTCT |  |
| E_H269188_c0_g1 | PLY | F: TGCCAATTTCAACAGATCAAGG |  |
|  |  | R: CTCCACCTGTTTGTCATTTCTCC |  |
| E_H262264_c1_g2 | STE1 | F: GTCTCATCAACGAACAGCTCCA |  |
|  |  | R: TCTCCATCAACCCAGTCACAGA |  |
| E_H261710_c1_g1 | FK | F: GCTGGAATGATGGGATGGC |  |
|  |  | R: ACAGCGCACAAAACAACTGG |  |
| E_H266229_c2_g2 | KYPC1 | F: TTGCCTCAACTGGGTATAATCCT |  |
|  |  | R: AGATGTTGCCAATGCTGTGC |  |
| E_H266229_c2_g4 | KYPC2 | F: CCGTGATCCAAAGCAACCTT |  |
|  |  | R: TTGTTAGAGGACTTTTCCCCATC |  |
| E_H266992_c0_g1 | BOR4 | F: CTGTGATGATTGCAGGGCTTTA |  |
|  |  | R: AATGGTATGCGGAAGGGTTCT |  |
| E_H263711_c2_g3 | GPAT6 | F: ATTAAGGCCGTGGCTCTGTC |  |
|  |  | R: CGTGTTGATTGCGATGGGTA |  |
| E_H257517_c7_g2 | FDH | F: CGTCACATTGTCCGAGCATC |  |
|  |  | R: TGTCCTCCGTCGTCTCAAGC |  |
| E_H264203_c1_g1 | ACEB | F: CTAAAGATGTAATCCCAGCGACC |  |
|  |  | R: CTTGAGGCAAGACTATGGAACG |  |
| E_H266562_c2_g3 | CS | F: AAGAACCCCTACCCCAACGT |  |
|  |  | R: CGAAACACCGAAGAGGACAGT |  |
| E_H256471_c3_g7 | CYP86A1 | F: GCTGGTTCTTCTGGCTCGTG |  |
|  |  | R: GGCTAATGCCGCTTTCAGG |  |
| E_H266302_c2_g1 | FAR4 | F: TTCTGCCATCAACTGGTCTGC |  |
|  |  | R: GGAATCCTTCCTCCGTGCA |  |
| E_H270153_c2_g1 | QOR | F: TGCTCAGTGGATGCTTCGAG |  |
|  |  | R: TGACGAGGTCCATGTTGCG |  |
| Reference gene | | F: CTGGTATGGCATTCCGTGTA |  |
|  |  | R: AACCACATCCTCTTCGGTGTA |  |
