# Supplementary material for: Comparative anatomical and transcriptomic analyses of the color variation of leaves in Aquilaria sinensis
Source: PeerJ. 2021 Jun 22;9:e11586. doi: 10.7717/peerj.11586 (PMC8231315; doi:10.7717/peerj.11586)
Supplement: Supplemental Information 10 [file peerj-09-11586-s010.docx]

**Table S10. Sequences of GS and NS.**

| Name | Gene accession | Sequence |
| --- | --- | --- |
| GS | MT548012 | **1** ATTGCAGAAT CCCGTGAACC ATCGAGTCTT TGAACGCAAG TTGCGCCCCA AGCCTTTGGG  **61** CCGAGGGCAC GTCTGCCTGG GTGTCACGCA TTGTAGCCCC CCACCCTCGT CGTAATGTCT  **121** GTGAGGGCTG TGTGGGGCTG ATACTGGCCT TCCCGTATGC ACAGCAATGC GGTTGGCCCA  **181** AATGGAGGAA CCCAGGGCGG TGTATGCCAT GATGAACGGT GGTGTGTGCT TAGCCTGCCG  **241** TCGTTAGAGC ATCATGCGCA TCATGCCCTT GAGGTATGTT TCGTGGACAA CCCCGGTGCA  **301** ATCATAGCGC GCATCGCGAC CCCAGGTCAG GCGGGAGCAC CCGCTGAGTT TAAGCATATC  **361** AATAAGCGGA GGAAAAGAAA CTTACCAGGA TTCCCCTAGT AACGGCGAGC GAACCGGGAA  **421** TAGCCCAGCT TGAAAATTGG TCGCCCTCGG CGTTCGAATT GTA |
|  | MT548013 | **1** ATTGCAGAAT CCCGTGAACC ATCGAGTCTT TGAACGCAAG TTGCGCCCCA AGCCTTTGGG  **61** CCGAGGGCAC GTCTGCCTGG GTGTCACGCA TTGTAGCCCC CCACCCTCGT CGTAATGTCT  **121** GTGAGGGCTG TGTGGGGCTG ATACTGGCCT TCCCGTATGC ACAGCAATGC GGTTGGCCCA  **181** AATGGAGGAA CCCAGGGCGG TGTATGCCAT GATGAACGGT GGTGTGTGCT TAGCCTGCCG  **241** TCGTTAGAGC ATCATGCGCA TCATGCCCTT GAGGTATGTT TCGTGGACAA CCCCGGTGCA  **301** ATCATAGCGC GCATCGCGAC CCCAGGTCAG GCGGGAGCAC CCGCTGAGTT TAAGCATATC  **361** AATAAGCGGA GGAAAAGAAA CTTACCAGGA TTCCCCTAGT AACGGCGAGC GAACCGGGAA  **421** TAGCCCAGCT TGAAAATTGG TCGCCCTCGG CGTTCGAATT GTA |
|  | MT548014 | **1** ATTGCAGAAT CCCGTGAACC ATCGAGTCTT TGAACGCAAG TTGCGCCCCA AGCCTTTGGG  **61** CCGAGGGCAC GTCTGCCTGG GTGTCACGCA TTGTAGCCCC CCACCCTCGT CGTAATGTCT  **121** GTGAGGGCTG TGTGGGGCTG ATACTGGCCT TCCCGTATGC ACAGCAATGC GGTTGGCCCA  **181** AATGGAGGAA CCCAGGGCGG TGTATGCCAT GATGAACGGT GGTGTGTGCT TAGCCTGCCG  **241** TCGTTAGAGC ATCATGCGCA TCATGCCCTT GAGGTATGTT TCGTGGACAA CCCCGGTGCA  **301** ATCATAGCGC GCATCGCGAC CCCAGGTCAG GCGGGAGCAC CCGCTGAGTT TAAGCATATC  **361** AATAAGCGGA GGAAAAGAAA CTTACCAGGA TTCCCCTAGT AACGGCGAGC GAACCGGGAA  **421** TAGCCCAGCT TGAAAATTGG TCGCCCTCGG CGTTCGAATT GTA |
|  | MT548018 | **1** AAGTGAGAAC TTTCAAATTC AGAGAAACCC TGGAATTAAA AACGGGCAAT CCTGAGCCAA  **61** ATCCTGTTTT ACAAAAATAA ACAAAGGTTC GGAAAGAAAG AATTCAAAAA AAAGGGGGGA  **121** TAGGTGCAGA GACTCAATGG AAGCTGTTCT AACAAATGGG GTTGACCACC TTTCCGTTAG  **181** TAAAGGAATC CTTCTATCGA AATTCCAGAA AGGATGAAGG ATAAACCTAT ACGCATACCT  **241** AAAGGTAATG AAAAACTATC TCAAAAAAGA GGACCAAAGC CCTTTTATAT TATATATTTT  **301** TATGAAAAAT AGAAATATAA AGAATTGTTG TGAATTGGTA TTGATTCCAA GTTGAAGAAA  **361** GAATCGATTA CAACATTCAT TAATAAAATT ATTCACCCCA AAGTTTGATA AATCTTTTGA  **421** AGAACGGATT AATCGGACGA GAATAAAGAT AGAGTCCCGT TCTACATGTC AATATCAATA  **481** CTGACAAGAA TGCAATTTAT AGTAAGAGGA AAATCCGTCG ACTTTGAAAA TCGTGAGGGT  **541** TCAAGTCCCT CTATCCCCAA CCTCCAAAAC CCGCTGACGC TCTACCTATT TTTTTTTTTT  **601** TGATTTCTAC CTATTTTTTA TCTTACCCTC TCCTTTTGTT AGGGGTTCAA AGTTACTTAT  **661** CTTTCCCATT CATCCAATTC TTTTCCATTT TACAGGCGTA TCCAAGCATC ATTTTTTCTC  **721** TTAACACAAA TATAGGTACA AATCAACATT TTTGAGTAAG GAATACCCAT TTGAATGATT  **781** CAAAATCATT ACTCAGACTG AAACTTACAT ACAAAGTCGT CCCTTCAAAG ATTCTGTCAA  **841** TTCCCGTTCG CTACTTTGAC TTTGATTTTA TTTTAATACT TTTTTTCGTC TTTTTTTTTT  **901** TCATTTTATT TTGATTTTTT CATTTTTAAT TGACATAGAC ACAAGTCCTC TATAAAAATG  **961** AGGATGATGT |
|  | MT548019 | **1** AAGTGAGAAC TTTCAAATTC AGAGAAACCC TGGAATTAAA AACGGGCAAT CCTGAGCCAA  **61** ATCCTGTTTT ACAAAAATAA ACAAAGGTTC GGAAAGAAAG AATTCAAAAA AAAGGGGGGA  **121** TAGGTGCAGA GACTCAATGG AAGCTGTTCT AACAAATGGG GTTGACCACC TTTCCGTTAG  **181** TAAAGGAATC CTTCTATCGA AATTCCAGAA AGGATGAAGG ATAAACCTAT ACGCATACCT  **241** AAAGGTAATG AAAAACTATC TCAAAAAAGA GGACCAAAGC CCTTTTATAT TATATATTTT  **301** TATGAAAAAT AGAAATATAA AGAATTGTTG TGAATTGGTA TTGATTCCAA GTTGAAGAAA  **361** GAATCGATTA CAACATTCAT TAATAAAATT ATTCACCCCA AAGTTTGATA AATCTTTTGA  **421** AGAACGGATT AATCGGACGA GAATAAAGAT AGAGTCCCGT TCTACATGTC AATATCAATA  **481** CTGACAAGAA TGCAATTTAT AGTAAGAGGA AAATCCGTCG ACTTTGAAAA TCGTGAGGGT  **541** TCAAGTCCCT CTATCCCCAA CCTCCAAAAC CCGCTGACGC TCTACCTATT TTTTTTTTTT  **601** TGATTTCTAC CTATTTTTTA TCTTACCCTC TCCTTTTGTT AGGGGTTCAA AGTTACTTAT  **661** CTTTCCCATT CATCCAATTC TTTTCCATTT TACAGGCGTA TCCAAGCATC ATTTTTTCTC  **721** TTAACACAAA TATAGGTACA AATCAACATT TTTGAGTAAG GAATACCCAT TTGAATGATT  **781** CAAAATCATT ACTCAGACTG AAACTTACAT ACAAAGTCGT CCCTTCAAAG ATTCTGTCAA  **841** TTCCCGTTCG CTACTTTGAC TTTGATTTTA TTTTAATACT TTTTTTCGTC TTTTTTTTTT  **901** TCATTTTATT TTGATTTTTT CATTTTTAAT TGACATAGAC ACAAGTCCTC TATAAAAATG  **961** AGGATGATGT |
|  | MT548020 | **1** AAGTGAGAAC TTTCAAATTC AGAGAAACCC TGGAATTAAA AACGGGCAAT CCTGAGCCAA  **61** ATCCTGTTTT ACAAAAATAA ACAAAGGTTC GGAAAGAAAG AATTCAAAAA AAAGGGGGGA  **121** TAGGTGCAGA GACTCAATGG AAGCTGTTCT AACAAATGGG GTTGACCACC TTTCCGTTAG  **181** TAAAGGAATC CTTCTATCGA AATTCCAGAA AGGATGAAGG ATAAACCTAT ACGCATACCT  **241** AAAGGTAATG AAAAACTATC TCAAAAAAGA GGACCAAAGC CCTTTTATAT TATATATTTT  **301** TATGAAAAAT AGAAATATAA AGAATTGTTG TGAATTGGTA TTGATTCCAA GTTGAAGAAA  **361** GAATCGATTA CAACATTCAT TAATAAAATT ATTCACCCCA AAGTTTGATA AATCTTTTGA  **421** AGAACGGATT AATCGGACGA GAATAAAGAT AGAGTCCCGT TCTACATGTC AATATCAATA  **481** CTGACAAGAA TGCAATTTAT AGTAAGAGGA AAATCCGTCG ACTTTGAAAA TCGTGAGGGT  **541** TCAAGTCCCT CTATCCCCAA CCTCCAAAAC CCGCTGACGC TCTACCTATT TTTTTTTTTT  **601** TGATTTCTAC CTATTTTTTA TCTTACCCTC TCCTTTTGTT AGGGGTTCAA AGTTACTTAT  **661** CTTTCCCATT CATCCAATTC TTTTCCATTT TACAGGCGTA TCCAAGCATC ATTTTTTCTC  **721** TTAACACAAA TATAGGTACA AATCAACATT TTTGAGTAAG GAATACCCAT TTGAATGATT  **781** CAAAATCATT ACTCAGACTG AAACTTACAT ACAAAGTCGT CCCTTCAAAG ATTCTGTCAA  **841** TTCCCGTTCG CTACTTTGAC TTTGATTTTA TTTTAATACT TTTTTTCGTC TTTTTTTTTT  **901** TCATTTTATT TTGATTTTTT CATTTTTAAT TGACATAGAC ACAAGTCCTC TATAAAAATG  **961** AGGATGATGT |
| NS | MT548015 | **1** TATGCGATTT ATTTGGTGTG AATTGCAGAA TCCCGTGAAC CATCGAGTCT TTGAACGCAA  **61** GTTGCGCCCC AAGCCTTTGG GCCGAGGGCA CGTCTGCCTG GGTGTCACGC ATTGTAGCCC  **121** CCCACCCTCG TCGTAATGTC TGTGAGGGCT GTGTGGGGCT GATACTGGCC TTCCCGTATG  **181** CACAGCAATG CGGTTGGCCC AAATGGAGGA ACCCAGGGCG GTGTATGCCA TGATGAACGG  **241** TGGTGTGTGC TTAGCCTGCC GTCGTTAGAG CATCATGCGC ATCATGCCCT TGAGGTATGT  **301** TTCGTGGACA ACCCCGGTGC AATCATAGCG CGCATCGCGA CCCCAGGTCA GGCGGGAGCA  **361** CCCGCTGAGT TTAAGCATAT CAATAAGCGG AGGAAAAGAA ACTTACCAGG ATTCCCCTAG  **421** TAACGGCGAG CGAACCGGGA ATAGCCCAGC TTGAAAATTG GTCGCCCTCG GCGTTCGAAT  **481** TGTAGTCTGA AAAAAACCGT C |
|  | MT548016 | **1** GTGTGAATTG CAGAATCCCG TGAACCATCG AGTCTTTGAA CGCAAGTTGC GCCCCAAGCC  **61** TTTGGGCCGA GGGCACGTCT GCCTGGGTGT CACGCATTGT AGCCCCCCAC CCTCGTCGTA  **121** ATGTCTGTGA GGGCTGTGTG GGGCTGATAC TGGCCTTCCC GTATGCACAG CAATGCGGTT  **181** GGCCCAAATG GAGGAACCCA GGGCGGTGTA TGCCATGATG AACGGTGGTG TGTGCTTAGC  **241** CTGCCGTCGT TAGAGCATCA TGCGCATCAT GCCCTTGAGG TATGTTTCGT GGACAACCCC  **301** GGTGCAATCA TAGCGCGCAT CGCGACCCCA GGTCAGGCGG GAGCACCCGC TGAGTTTAAG  **361** CATATCAATA AGCGGAGGAA AAGAAACTTA CCAGGATTCC CCTAGTAACG GCGAGCGAAC  **421** CGGGAATAGC CCAGCTTGAA AATTGGTCGC CCTCGGCGTT CGAATTG |
|  | MT548017 | **1** GTGTGAATTG CAGAATCCCG TGAACCATCG AGTCTTTGAA CGCAAGTTGC GCCCCAAGCC  **61** TTTGGGCCGA GGGCACGTCT GCCTGGGTGT CACGCATTGT AGCCCCCCAC CCTCGTCGTA  **121** ATGTCTGTGA GGGCTGTGTG GGGCTGATAC TGGCCTTCCC GTATGCACAG CAATGCGGTT  **181** GGCCCAAATG GAGGAACCCA GGGCGGTGTA TGCCATGATG AACGGTGGTG TGTGCTTAGC  **241** CTGCCGTCGT TAGAGCATCA TGCGCATCAT GCCCTTGAGG TATGTTTCGT GGACAACCCC  **301** GGTGCAATCA TAGCGCGCAT CGCGACCCCA GGTCAGGCGG GAGCACCCGC TGAGTTTAAG  **361** CATATCAATA AGCGGAGGAA AAGAAACTTA CCAGGATTCC CCTAGTAACG GCGAGCGAAC  **421** CGGGAATAGC CCAGCTTGAA AATTGGTCGC CCTCGGCGTT CGAATTG |
|  | MT548021 | **1** CTTAGTATGG AACCTACTAA GTGAGAACTT TCAAATTCAG AGAAACCCTG GAATTAAAAA  **61** CGGGCAATCC TGAGCCAAAT CCTGTTTTAC AAAAATAAAC AAAGGTTCGG AAAGAAAGAA  **121** TTCAAAAAAA AGGGGGGATA GGTGCAGAGA CTCAATGGAA GCTGTTCTAA CAAATGGGGT  **181** TGACCACCTT TCCGTTAGTA AAGGAATCCT TCTATCGAAA TTCCAGAAAG GATGAAGGAT  **241** AAACCTATAC GCATACCTAA AGGTAATGAA AAACTATCTC AAAAAAGAGG ACCAAAGCCC  **301** TTTTATATTA TATATTTTTA TGAAAAATAG AAATATAAAG AATTGTTGTG AATTGGTATT  **361** GATTCCAAGT TGAAGAAAGA ATCGATTACA ACATTCATTA ATAAAATTAT TCACCCCAAA  **421** GTTTGATAAA TCTTTTGAAG AACGGATTAA TCGGACGAGA ATAAAGATAG AGTCCCGTTC  **481** TACATGTCAA TATCAATACT GACAAGAATG CAATTTATAG TAAGAGGAAA ATCCGTCGAC  **541** TTTGAAAATC GTGAGGGTTC AAGTCCCTCT ATCCCCAACC TCCAAAACCC GCTGACGCTC  **601** TACCTATTTT TTTTTTTTGA TTTCTACCTA TTTTTTATCT TACCCTCTCC TTTTGTTAGT  **661** GGTTCAAAGT TACTTATCTT TCCCATTCAT CCAATTCTTT TCCATTTTAC AGGCGTATCC  **721** AAGCATCATT TTTTCTCTTA ACACAAATAT AGGTACAAAT CAACATTTTT GAGTAAGGAA  **781** TACCCATTTG AATGATTCAA AATCATTACT CAGACTGAAA CTTACATACA AAGTCGTCCC  **841** TTCAAAGATT CTGTCAATTC CCGTTCGCTA CTTTGACTTT GATTTTATTT TAATACTTTT  **901** TTTCGTCTTT TTTTTTTTCA TTTTATTTTG ATTTTTTCAT TTTTAATTGA CATAGACACA  **961** AGTCCTCTAT AAAAATGAGG ATGATGTCTT GGTAATGGTC GGGATAGCTC AGC |
|  | MT548022 | **1** AGCTGAGCTA TCCCGACCAT TACCAAGACA TCATCCTCAT TTTTATAGAG GACTTGTGTC  **61** TATGTCAATT AAAAATGAAA AAATCAAAAT AAAATGAAAA AAAAAAAGAC GAAAAAAAGT  **121** ATTAAAATAA AATCAAAGTC AAAGTAGCGA ACGGGAATTG ACAGAATCTT CGAAGGGACG  **181** ACTTTGTATG TAAGTTTCAG TCTGAGTAAT GATTTTGAAT CATTCAAATG GGTATTCCTT  **241** ACTCAAAAAT GTTGATTTGT ACCTATATTT GTGTTAAGAG AAAAAATGAT GCTCGGATAC  **301** GCCTGTAAAA TGGAAAAGAA TAGGATGAAT GGGAAAGATA AGTAACTTTG AACCACTAAC  **361** AAAAGGAGAG GGTAAGATAA AAAATAGGTA GAAATCAAAA AAAAAAAATA GGTAGAGCGT  **421** CAGCGGGTTT TGGAGGTTGG GGATAGAGGG ACTTGAACCC TCACGATTTT CAAAGTCGAC  **481** GGATTTTCCT CTTACTATAA ATTGCATTCT TGTCAGTATT GATATTGACA TGTAGAACGG  **541** GACTCTATCT TTATTCTCGT CCGATTAATC CGTTCTTCAA AAGATTTATC AAACTTTGGG  **601** GTGAATAATT TTATTAATGA ATGTTGTAAT CGATTCTTTC TTCAACTTGG AATCAATACC  **661** AATTCACAAC AATTCTTTAT ATTTCTATTT TTCATAAAAA TATATAATAT AAAAGGGCTT  **721** TGGTCCTCTT TTTTGAGATA GTTTTTCATT ACCTTTAGGT ATGCGTATAG GTTTATCCTT  **781** CATCCTTTCT GGAATTTCGA TAGAAGGATT CCTTTACTAA CGGAAAGGTG GTCAACCCCA  **841** TTTGTTAGAA CAGCTTCCAT TGAGTCTCTG CACCTATCCC CCCTTTTTTT TGAATTCTTT  **901** CTTTCCGAAC CTTTGTTTAT TTTTGTAAAA CAGGATTTGG CTCAGGATTG CCCGTTTTTA  **961** ATTCCAGGGT TTCTCTGAAT TTGAAAGTTC TCACTTAGTA GGTTCCATAC TAAG |
|  | MT548023 | **1** ATCCTCATTT TTATAGAGGA CTTGTGTCTA TGTCAATTAA AAATGAAAAA ATCAAAATAA  **61** AATGAAAAAA AAAAAGACGA AAAAAAGTAT TAAAATAAAA TCAAAGTCAA AGTAGCGAAC  **121** GGGAATTGAC AGAATCTTCG AAGGGACGAC TTTGTATGTA AGTTTCAGTC TGAGTAATGA  **181** TTTTGAATCA TTCAAATGGG TATTCCTTAC TCAAAAATGT TGATTTGTAC CTATATTTGT  **241** GTTAAGAGAA AAAATGATGC TCGGATACGC CTGTAAAATG GAAAAGAATA GGATGAATGG  **301** GAAAGATAAG TAACTTTGAA CCACTAACAA AAGGAGAGGG TAAGATAAAA AATAGGTAGA  **361** AATCAAAAAA AAAAAAATAG GTAGAGCGTC AGCGGGTTTT GGAGGTTGGG GATAGAGGGA  **421** CTTGAACCCT CACGATTTTC AAAGTCGACG GATTTTCCTC TTACTATAAA TTGCATTCTT  **481** GTCAGTATTG ATATTGACAT GTAGAACGGG ACTCTATCTT TATTCTCGTC CGATTAATCC  **541** GTTCTTCAAA AGATTTATCA AACTTTGGGG TGAATAATTT TATTAATGAA TGTTGTAATC  **601** GATTCTTTCT TCAACTTGGA ATCAATACCA ATTCACAACA ATTCTTTATA TTTCTATTTT  **661** TCATAAAAAT ATATAATATA AAAGGGCTTT GGTCCTCTTT TTTGAGATAG TTTTTCATTA  **721** CCTTTAGGTA TGCGTATAGG TTTATCCTTC ATCCTTTCTG GAATTTCGAT AGAAGGATTC  **781** CTTTACTAAC GGAAAGGTGG TCAACCCCAT TTGTTAGAAC AGCTTCCATT GAGTCTCTGC  **841** ACCTATCCCC CCTTTTTTTT GAATTCTTTC TTTCCGAACC TTTGTTTATT TTTGTAAAAC  **901** AGGATTTGGC TCAGGATTGC CCGTTTTTAA TTCCAGGG |
