# Supplementary figures and images for: Comparative anatomical and transcriptomic analyses of the color variation of leaves in Aquilaria sinensis
Source: PeerJ. 2021 Jun 22;9:e11586. doi: 10.7717/peerj.11586 (PMC8231315; doi:10.7717/peerj.11586)

ITS2

GS\_1

GS\_2

GS\_3

NS\_1

NS\_2

NS\_3

2000 bp

1000 bp

750 bp

500 bp

250 bp

100 bp

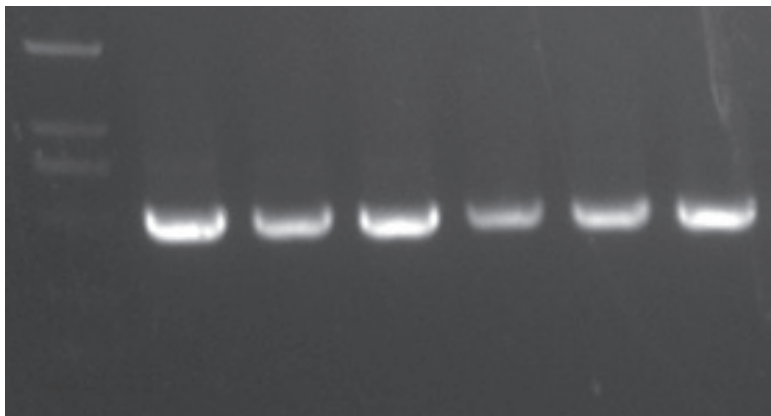

trnL-trnF

GS\_1

GS\_2

GS\_3

NS\_1

NS\_2

NS\_3

2000 bp

1000 bp

750 bp

500 bp

250 bp

100 bp

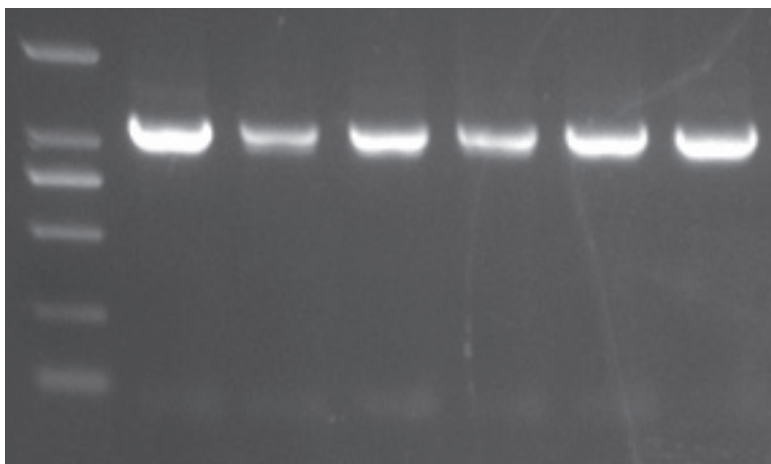

Supplement: Supplemental Information 13 [file peerj-09-11586-s013.pdf]

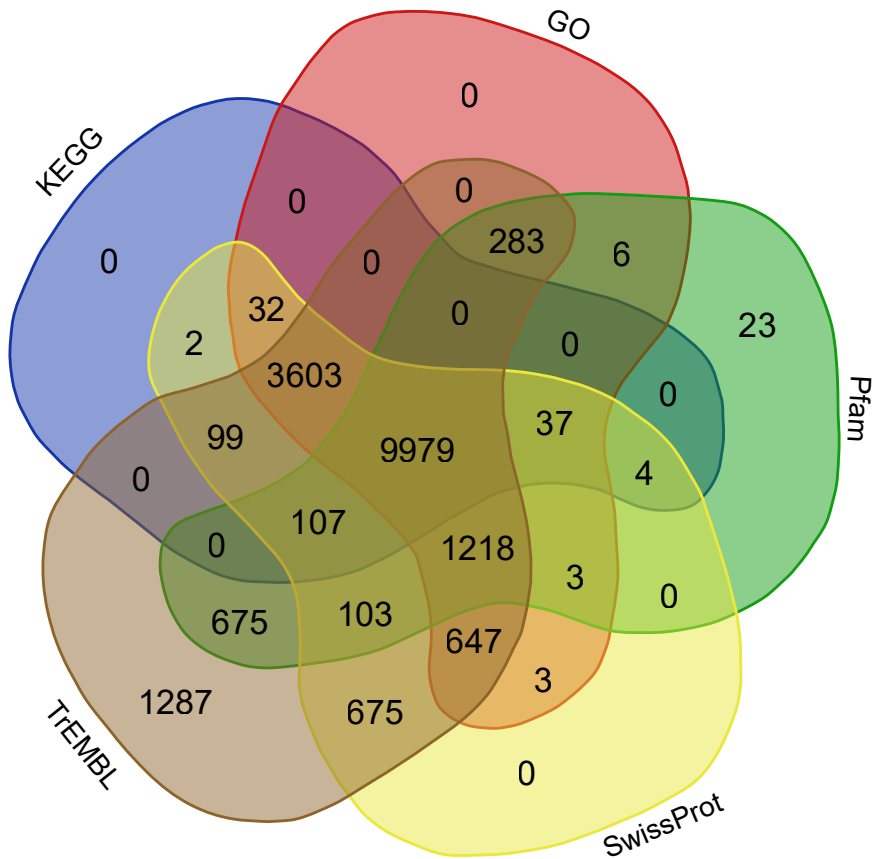

Supplement: Supplemental Information 15 [file peerj-09-11586-s015.pdf]

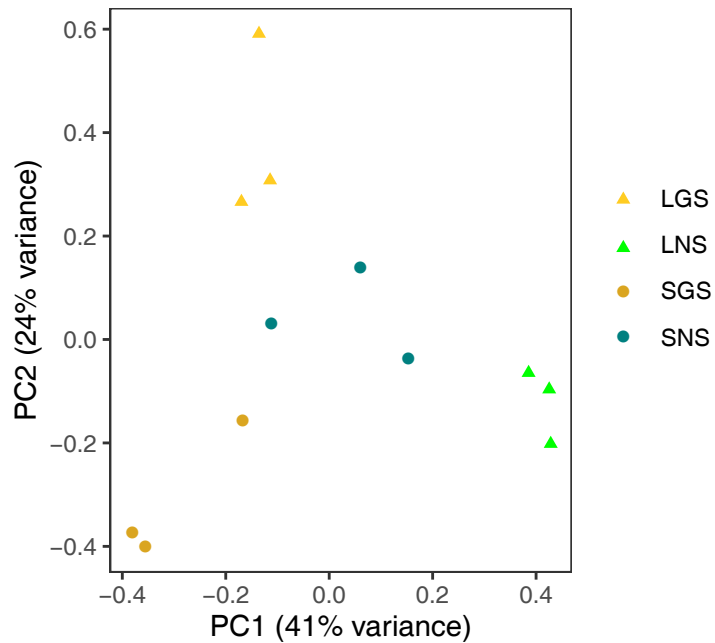

Supplement: Supplemental Information 16 [file peerj-09-11586-s016.pdf]

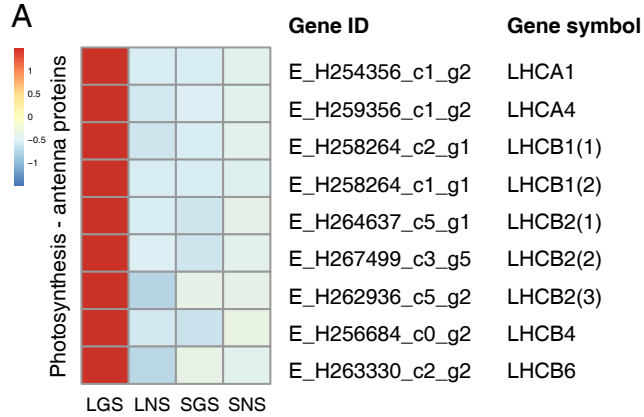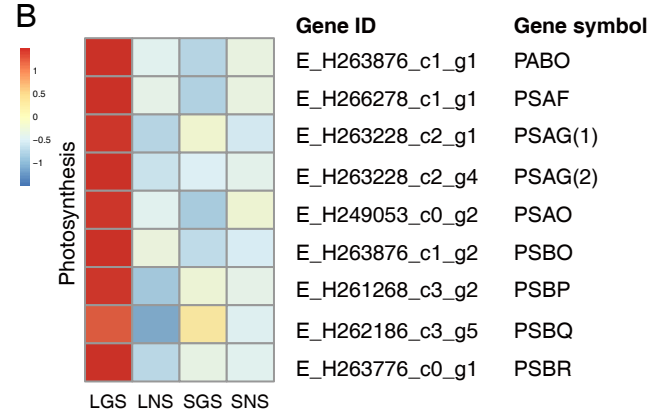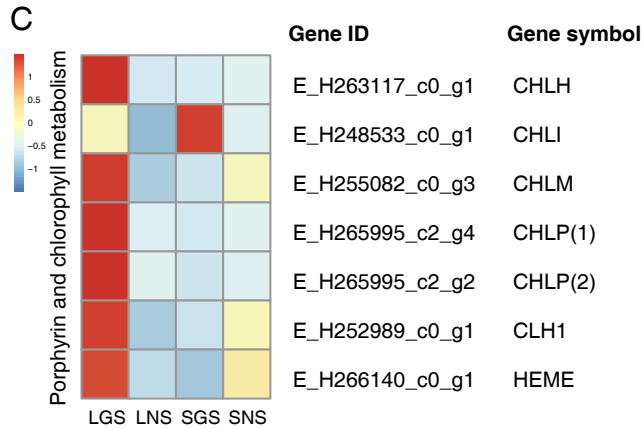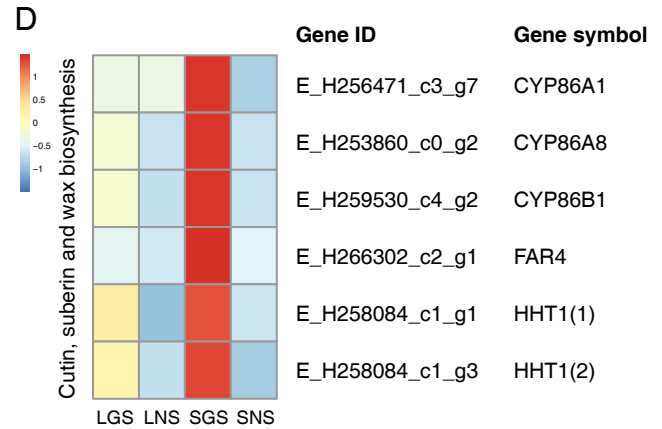

Supplement: Supplemental Information 18 — The color of each cell is on behalf of the Z-score indicating the relative gene expression. [file peerj-09-11586-s018.pdf]
