# Supplementary material for: Comparative anatomical and transcriptomic analyses of the color variation of leaves in Aquilaria sinensis
Source: PeerJ. 2021 Jun 22;9:e11586. doi: 10.7717/peerj.11586 (PMC8231315; doi:10.7717/peerj.11586)
Supplement: Supplemental Information 14 [file peerj-09-11586-s014.pdf]

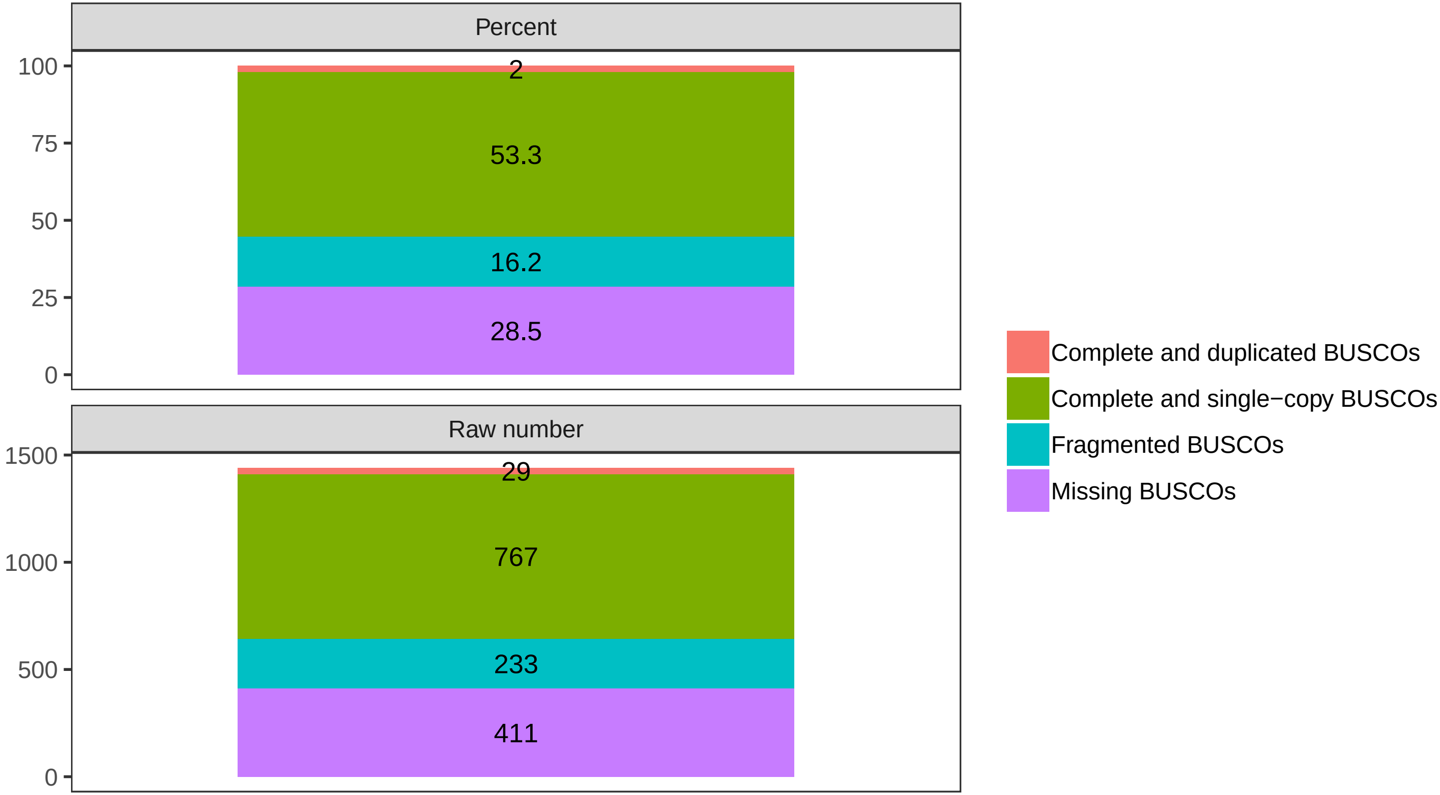
Percent
100
75
50
25
0
2
53.3
16.2
28.5
Raw number
1500
1000
500
0
29
767
233
411
Complete and duplicated BUSCOs
Complete and single−copy BUSCOs
Fragmented BUSCOs
Missing BUSCOs
