# Supplementary material for: Comparative anatomical and transcriptomic analyses of the color variation of leaves in Aquilaria sinensis
Source: PeerJ. 2021 Jun 22;9:e11586. doi: 10.7717/peerj.11586 (PMC8231315; doi:10.7717/peerj.11586)
Supplement: Supplemental Information 17 — Log2 (fold change) is computed using their average abundance in golden samples dividing them in normal samples. [file peerj-09-11586-s017.pdf]

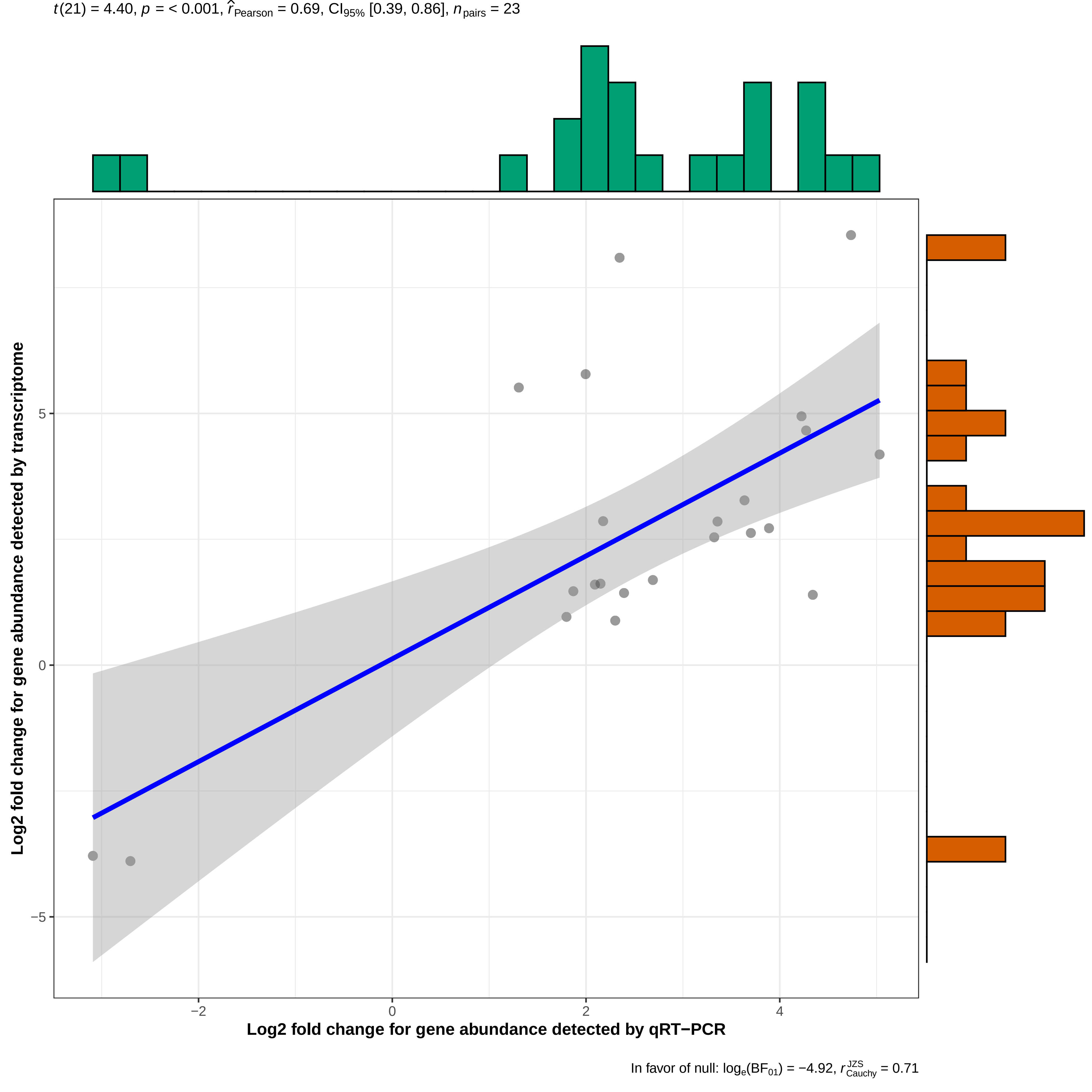
t(21) = 4.40, p = < 0.001, r̂Pearson = 0.69, CI95% [0.39, 0.86], npairs = 23
Log2 fold change for gene abundance detected by transcriptome
Log2 fold change for gene abundance detected by qRT−PCR
5
0
−5
−2
0
2
4
In favor of null: loge(BF01) = −4.92, rJZS Cauchy = 0.71
